# FMM-AutoOpt: A Python Tool for Automated GROMACS FMM Parameter Optimization

**DOI:** 10.1021/acs.jcim.6c01211

**Published:** 2026-09-04

**Authors:** Louise J. Persson, Erik G. Marklund

**Affiliations:** Department of Chemistry for Life Sciences, 8097 Uppsala University, SE-75123 Uppsala, Sweden

## Abstract

The fast multipole method (FMM) for computing Coulombic forces in molecular dynamics (MD) simulations offers benefits for a variety of systems, including nonperiodic configurations such as molecules in vacuum and large-scale systems where many other approaches encounter limitations. Because FMM parameters govern both simulation accuracy and computational performance, and because the optimal solution is highly system-dependent, we have developed FMM-AutoOpt for the automated benchmarking of FMM parameters with GROMACS. Relying solely on standard Python libraries and GROMACS FMM ( KohnkeB.,  J. Chem. Theory Comput. 2020, 16, 6938–6949 10.1021/acs.jctc.0c00744PMC766074633084336), the tool is versatile and flexible, facilitating rapid parameter screening to ensure optimal performance and high simulation accuracy.

## Graphical abstract

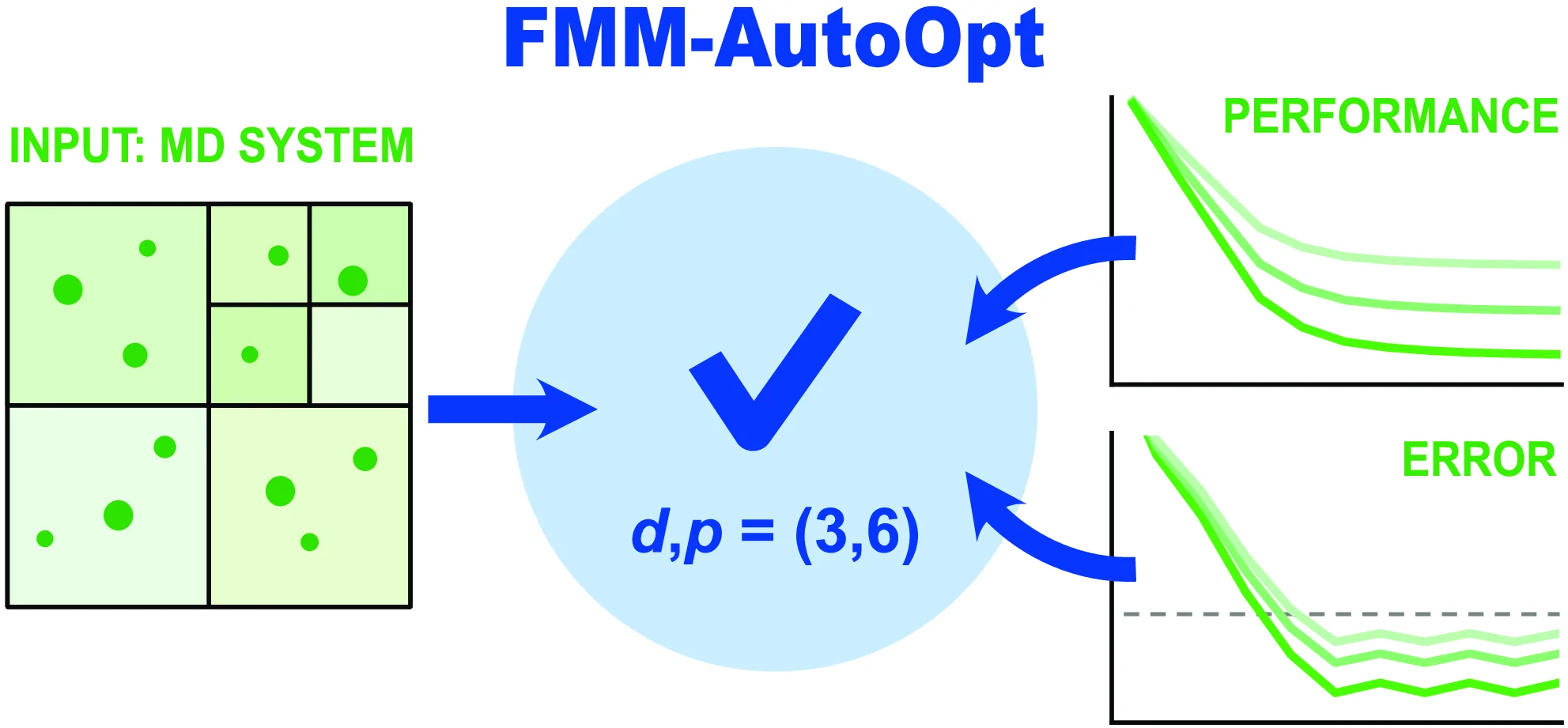

## Introduction

The FMM algorithm for efficient computation of long-range forces in particle simulations was first described by Greengard and Rokhlin in 1987.^1^ By relying on an octree subdivision scheme combined with truncated multipole expansions to describe particle distributions, the method avoids the explicit computation of the pairwise interactions. However, despite its clear utility for Coulomb force computation in MD simulations, most MD software packages have favored other methods.^2^ Perhaps the most widely adopted of these is the particle mesh Ewald (PME),^3^ which provides high computational performance and accuracy for many commonly simulated systems, particularly condensed-phase systems of moderate to large scales.

Recently, Kohnke et al.^4,5^ implemented FMM with the widely used MD package GROMACS,^6^ including an extension to constant-pH simulations.^7,8^ FMM offers several advantages over PME across a variety of applications. First, FMM achieves *O*(*N*) scaling with the number of particles,^1,9^ in comparison to PME’s *O*(*N* log *N*). FMM also exhibits superior multinode scaling properties, offering potential to speed up computations of ultra-large systems. These simulations are becoming increasingly relevant as computing capacities expand. Second, FMM supports nonperiodic systems. In contrast, PME is based on Ewald summation, which assumes periodic boundary conditions (PBCs). Consequently, PME is only strictly accurate for systems with a neutral net charge, a constraint that does not apply to FMM. Third, FMM more efficiently describes sparse systems. PME relies on a regular grid to describe the charge distribution across the entire simulation box, which must be sufficiently dense to ensure an accurate solution. Through octree subdivision, FMM can identify and ignore empty boxes within sparse systems, optimizing the computational effort.

GROMACS FMM has been benchmarked for single water droplets,^4^ aerosols,^4^ and gas-phase proteins.^9^ These studies have highlighted the necessity of optimizing the FMM parameters on a system-specific basis. The tree depth *d* and the multipole order *p* govern both the accuracy of the Coulomb force computation and the computational performance, and their optimal settings can vary drastically depending on the system size and particle distribution. For example, we previously demonstrated that linear scaling for gas-phase proteins can only be achieved if these parameters are properly optimized.^9^

Here, we present FMM-AutoOpt, a highly automated Python tool that systematically evaluates FMM parameters for a given molecular system to facilitate the determination of optimized simulation settings. The tool benchmarks accuracy and computational performance across a target range of (*d*, *p*) parameter combinations and can also assess the use of the FMM_SPARSE environment variable, which enables empty boxes to be skipped for potentially enhanced performance. The underlying approach of the tool is similar to the benchmarks performed in our previous work;^9^ however, a key distinction in this implementation is that accuracy testing is based on the error in Coulombic energy of the system rather than the average error in Coulombic force per atom. This modification allows the tool to run solely within the GROMACS FMM environment without dependency on non-standard Python packages. FMM-AutoOpt is freely available from https://github.com/lpersson/FMM-AutoOpt.git.

## Algorithm

### Input

The required inputs are one or more GROMACS structure files (.gro) and one corresponding topology file (.top). FMM-AutoOpt performs short simulations across a range of FMM parameters to directly evaluate the accuracy and computational performance of a given system. To ensure stable trajectories, it is recommended that the input structure undergo energy minimization and be sufficiently equilibrated to the simulation conditions.

The current version of GROMACS FMM was implemented specifically for cubic geometries, and accuracy may be compromised when using noncubic boxes.^4^ FMM-AutoOpt generates a cubic box with a minimum distance of 3 nm between the solute and the box, unless the provided .gro file already defines a cubic box.

Default simulation parameters are supplied for a standard simulation of gas-phase molecules with virtual sites^10^ (Table 1). Optionally, users may provide their own parameter file (.mdp), making the tool compatible with any simulation type or system. Certain parameters are necessarily overridden to meet the specific requirements of the test design, see Figure 1. The environment variables OPENBOUNDARY and FMM_SPARSE can be set by the user as inputs to FMM-AutoOpt. If not defined, then they are automatically set to 1 and 0, respectively. Other environmental variables must be set by the user before running the program.

**Figure 1:**
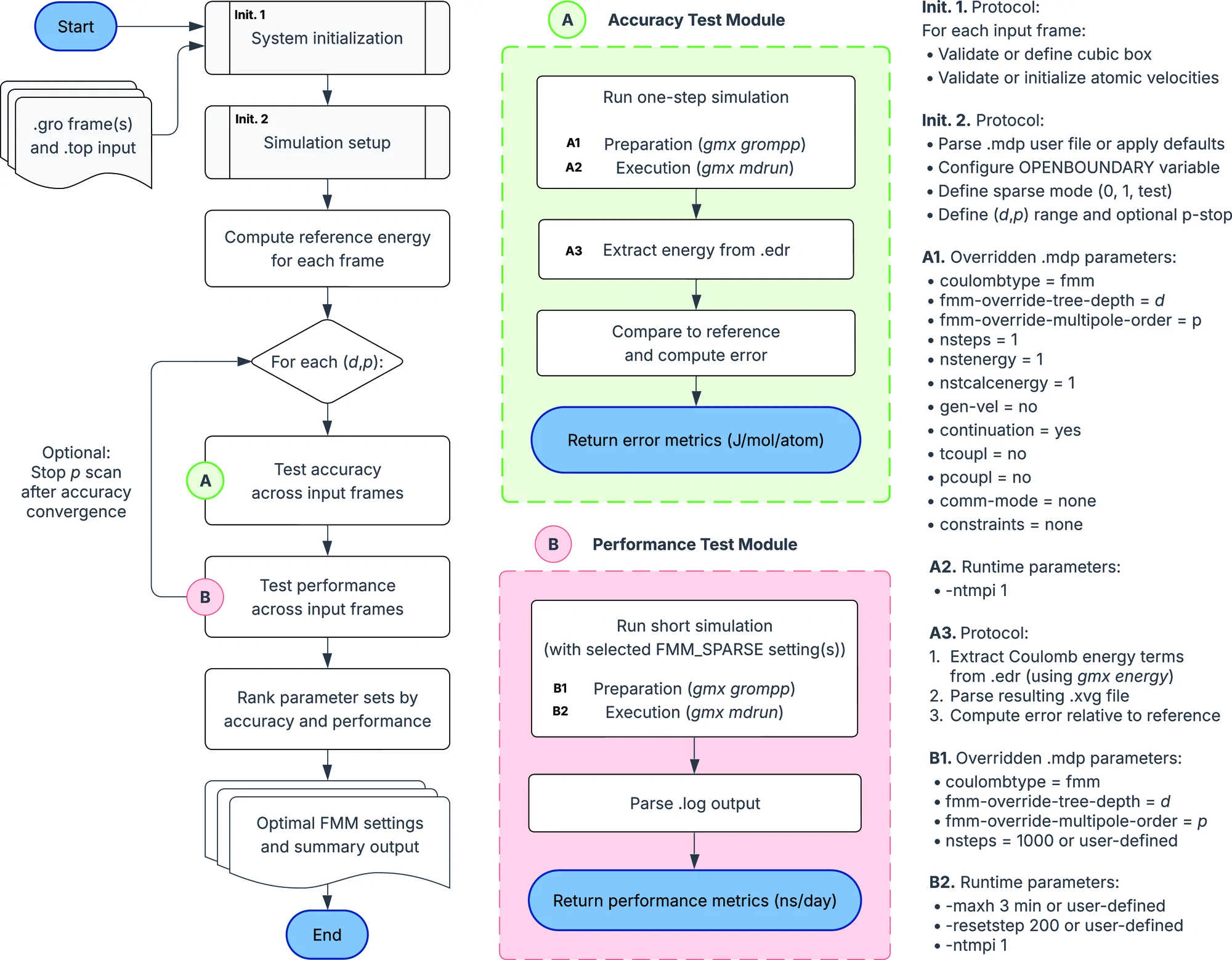
Flowchart outlining the main steps and components of FMM-AutoOpt. The tool is built around two test modules, and relies on commands within the GROMACS FMM^4^ environment.

**Table 1:** Default Simulation Parameters; Adapted for Simulation of Gas-Phase Proteins

option	setting
integrator	md
dt	0.005
comm-mode	angular
nstcomm	10
verlet-buffer-tolerance	–1
rlist	1
coulombtype	fmm
tcoupl	v-rescale
tc-grps	system
ref-t	300
tau-t	0.1
constraints	all-bonds
constraint-algorithm	lincs
lincs-order	6
lincs-iter	1

The ranges of *d* and *p* to be evaluated can be specified by the user. If these ranges are not provided, they are set to *d* ∈ [2, 4], *p* ∈ [0, 20], using a step size of 2 for *p*. These defaults cover the range relevant to most simulations.

### Test Modules and Output

As outlined in Figure 1, FMM-AutoOpt comprises two distinct test modules: an accuracy test (A) and a performance test (B). The tool iterates through all specified (*d*, *p*) combinations to generate direct comparative data.

- Accuracy Test. The accuracy test relies on extracting the Coulomb energy from single-point energy evaluations compared to a reference solution, which is also obtained with FMM. For nonperiodic configurations, the exact solution can be obtained via direct pairwise summation, set as the default (*d*, *p*) = (0, 0) reference. For periodic systems, an exact direct calculation is not feasible. Instead, the reference is computed using FMM with user-selectable (*d*, *p*) parameters chosen to provide a sufficiently high accuracy. This flexibility allows the reference calculation to be tailored to the computational limits of specific systems and hardware.
  Furthermore, to ensure consistency in the error calculation, these evaluations utilize identical initial velocities; if the input .gro file lacks velocity data, then FMM-AutoOpt generates them automatically during a preparatory one-step simulation. Additionally, algorithms that modulate coordinates or velocities, such as barostats and bond constraints, are disabled during these tests to ensure that the reported errors strictly reflect those of the FMM force evaluation. Error values are reported in J/mol/atom.
- Performance Test. For performance benchmarking, simulations are executed with a defined maximum number of steps (--nsteps) and a wall-time limit (--maxh). To prevent initial startup overhead from impacting the results, the GROMACS performance counter is reset after a specified interval by using the --resetstep flag. These parameters default to standard values but can be manually tuned by the user in accordance with the size of the system and the desired measurement accuracy. To reduce the overall benchmarking time, the --p-stop option can be enabled to terminate the scan over *p* values for a given *d* once a stable accuracy convergence has been reached. The final performance data are extracted from the GROMACS .log file and reported in ns/day.

Because the accuracy and performance of FMM depend on the spatial distribution of atoms, structural changes during a simulation can influence the optimal choice of the parameters. FMM-AutoOpt therefore accepts multiple .gro files as input, for example, frames sampled from a preliminary trajectory, and repeats both accuracy and performance testing for each structure. This feature can also be used to postvalidate the accuracy of a completed simulation. Optionally, the FMM_SPARSE environment variable, which enables empty octree boxes to be skipped during the FMM calculation, can also be evaluated to determine which setting provides the best performance. This functionality is enabled with --sparse test, which repeats the performance benchmark for each (*d*, *p*) combination with FMM_SPARSE set to 0 and 1, respectively.

Upon completion of the benchmarking suite, the calculated errors and recorded performance metrics were summarized in output files. The tool also identifies and prints the highest-performing parameter combination that meets the accuracy threshold across all evaluated frames as well as near-convergence parameters, which can be useful for handling outliers when assessing multiple frames. This threshold can be defined by the user; otherwise, a default of 0.02 J/mol/atom is applied, based on empirical convergence trends observed in the benchmarks done on proteins in vacuum, detailed in the subsequent section. The default threshold should therefore be regarded as only a starting point. Users are strongly encouraged to determine an appropriate convergence threshold and to initially benchmark a sufficiently broad range of (*d*, *p*) values for each new system before restricting the search, since the absolute error at convergence is highly system-dependent.

## Benchmarks

The following benchmarks were performed with the same installation and hardware as described in ref 9. These results were obtained using the original GROMACS FMM implementation,^4,5^ though validity tests were also done with the constant-pH version^7,8^ (but without constant-pH applied) and on a variety of hardware.

### Proteins in Vacuum

To validate the fidelity of FMM-AutoOpt and the new energy-based error metric, we benchmarked two gas-phase protein systems previously used in our FMM study:^9^ concanavalin A (ConA, 100 kDa, PDB ID 1AZD^11^) and a HSP60/HSP10 heat shock protein complex (HSP, 940 kDa, PDB ID 6HT7 ^12^). Like previously, we employed the OPLSA-AA force field,^13^ virtual sites for hydrogens, and the previously described charge distributions. FMM-AutoOpt was run using its default settings, and performance and accuracy were evaluated across five frames extracted from a 1 ns simulation in vacuum, generated using FMM with the high-accuracy parameters identified in our previous benchmarks.

The results are shown in Figure 2. Using the total Coulomb energy of the system rather than the per-atom Coulombic force error employed in our previous benchmarks introduces a potential limitation. Because the metric averages over all atoms, partial error cancellation may occur, resulting in an increased stochasticity. Nevertheless, the accuracy profiles generated by FMM-AutoOpt closely reproduced our previous results, with convergence achieved at similar (*d*, *p*) parameter combinations (Figure 2). Furthermore, both proteins, despite differing substantially in size, reached a convergence below 0.02 J/mol/atom. This supports the choice of 0.02 J/mol/atom as the default accuracy threshold in FMM-AutoOpt, consistent with the other default settings being tailored to this kind of simulation.

**Figure 2:**
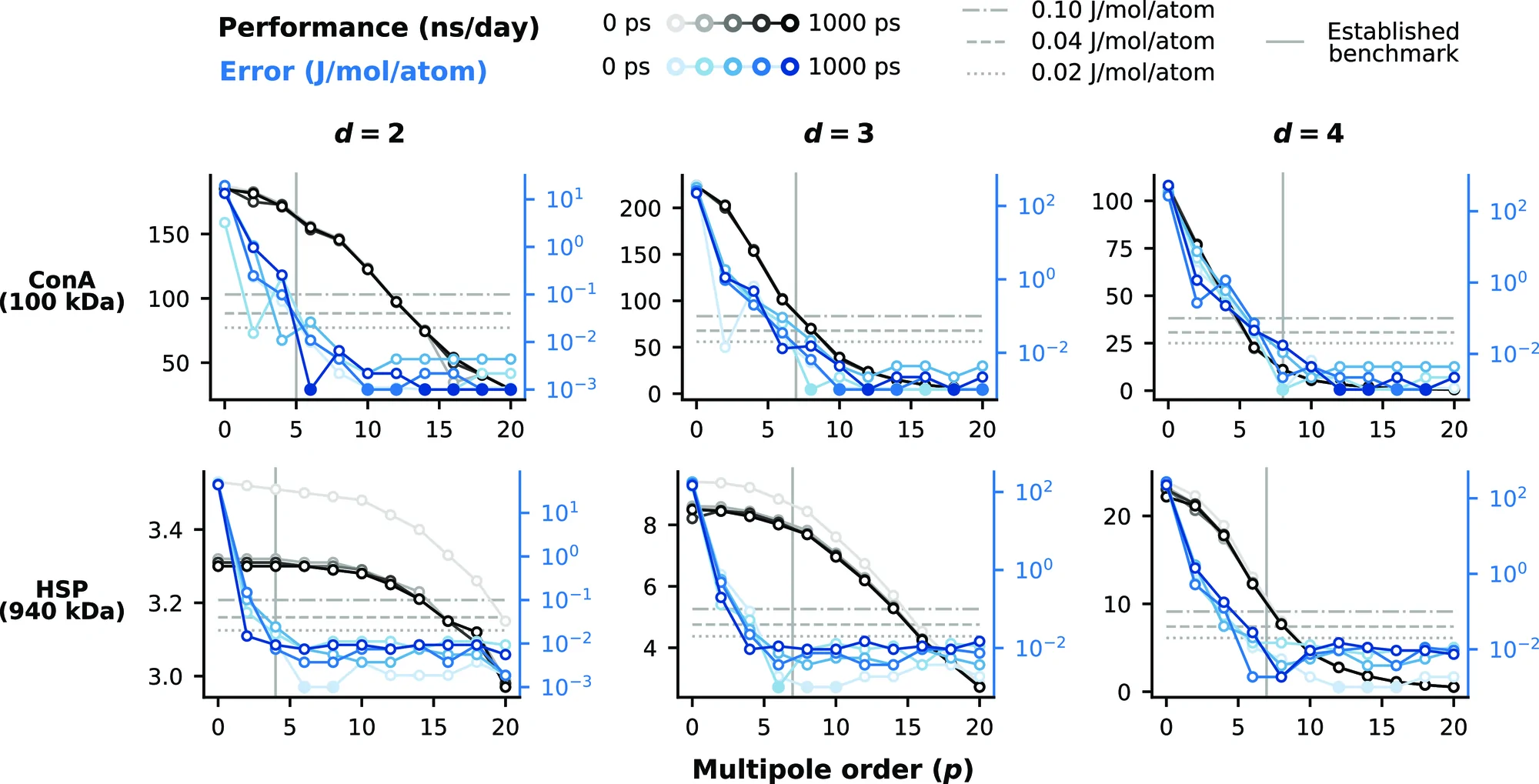
Benchmarks for two proteins in vacuum, performed with FMM-AutoOpt using default parameters and evaluated across five frames extracted from a 1000 ps equilibration trajectory. Solid markers denote zero error; these are plotted at 0.001 J/mol/atom for compatibility with the logarithmic scale. “Established benchmark” refers to parameter combinations where *p* at or above the plotted value yielded errors below the accuracy threshold in previous benchmarks.^9^

The benchmarks also demonstrate the utility of evaluating multiple-input frames. Repeating the analysis across frames not only provides replicate measurements, thereby improving the statistical robustness of the energy-based error metric but also accounts for structural changes occurring during the simulation. For the vacuum protein simulations considered here, the compaction induced by vacuum exposure had only a minor, and in many cases negligible, effect on the FMM accuracy and performance for a given set of parameters.

### Solvated Systems

We benchmarked three classes of solvated systems: the alcohol dehydrogenase protein complex (ADH, 150 kDa, PDB ID 2HCY), the Dickerson-Drew dodecameric DNA (PDB ID 4C64 ^14^), and aqueous NaCl solutions at concentrations of 200 mM and 2 M (Figures 3 and S2). The protein and DNA systems were solvated in cubic boxes with a minimum distance of 1 nm between the solute and box edges, whereas the salt solutions were simulated in cubic boxes with side lengths of 10 nm. All systems employed the TIP3P water model,^15^ and virtual sites for hydrogens were used only for the protein. The protein and salt solution systems were simulated with a 5 fs time step, whereas the DNA simulations used a 2 fs time step. The OPLS-AA force field was used for the protein and salt solutions, while the DNA simulations employed the OL15 force field.^16^ PBCs were applied in all directions, and the OPENBOUNDARY variable was set to 0. A custom .mdp file adapted for condensed-phase benchmarks was supplied to FMM-AutoOpt using temperature coupling but no pressure coupling. For comparison, ADH was also simulated in vacuum using the default settings, also maintaining a minimum 1 nm distance between the protein and the box boundaries.

**Figure 3:**
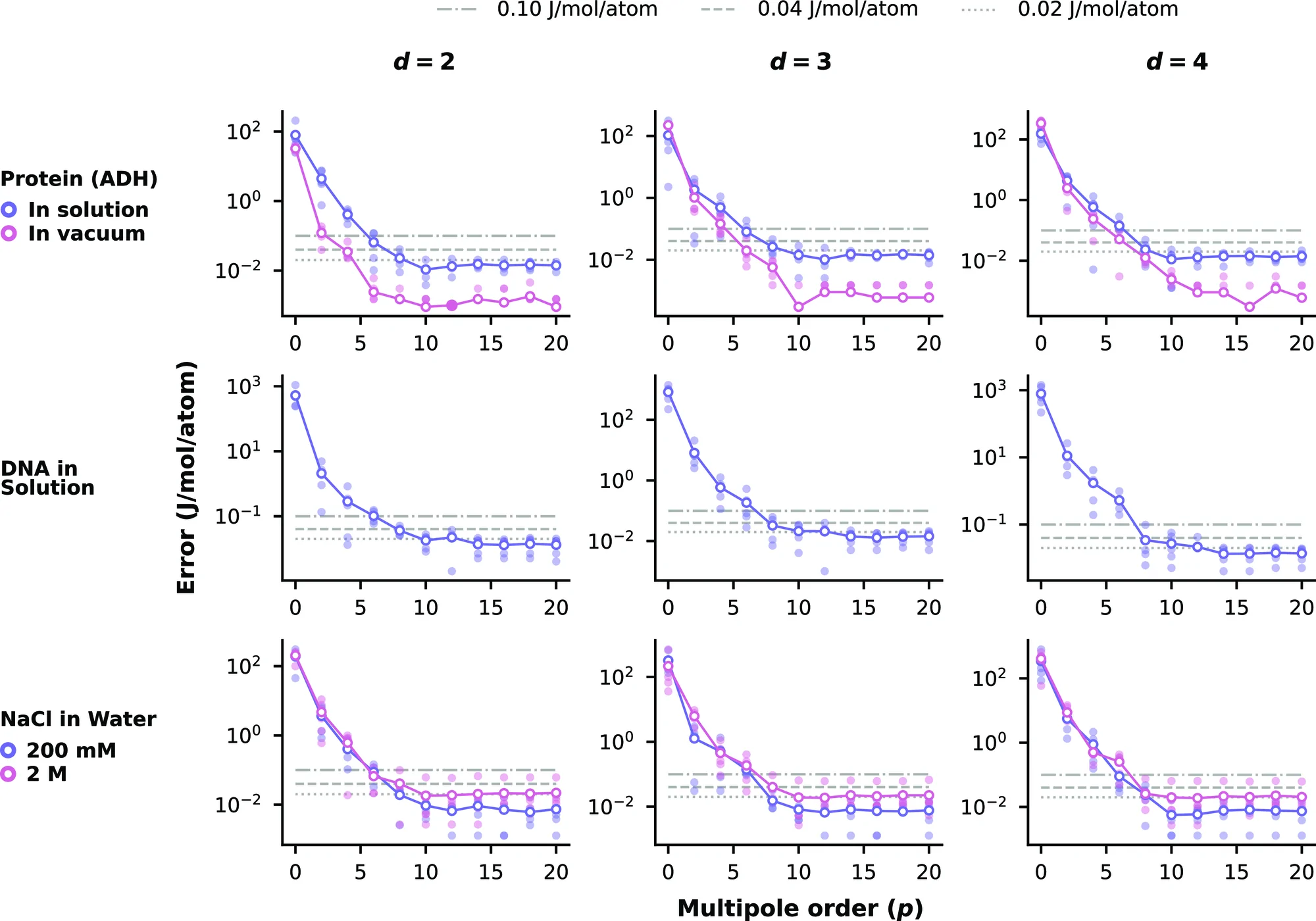
Accuracy benchmarks of solvated systems. Filled markers represent the mean values across five input frames, while semi-transparent markers denote the individual frame measurements. Solid markers denote zero mean error; these are plotted at 0.001 J/mol/atom for compatibility with the logarithmic scale. Computational details are given in the text and corresponding performance data are shown in Figure S2.

As an exact solution for the Coulomb energy cannot be calculated with PBCs, (*d*, *p*) = (0, 20) was used as a reference for accuracy testing, representing the highest-accuracy setting permitted by our hardware. Previous work has shown that, in single precision, the FMM accuracy reaches its limit at approximately *p* = 20 across tree depths and achieves an accuracy comparable to that of PME using high-accuracy settings.^4^ As an additional validation, we examined the convergence behavior of the reference calculations, providing further support for the use of (*d*, *p*) = (0, 20) as the reference state (Figure S1).

The benchmarks showed that solvated systems generally reached convergence at slightly higher absolute errors than the gas-phase systems (Figure 3). Nevertheless, the corresponding FMM parameter combinations were remarkably consistent. For example, solvated and gas-phase ADH converged at similar (*d*, *p*) values. Likewise, increasing the NaCl concentration increased the absolute error but had little effect on the convergence trend, and the DNA system exhibited a convergence behavior highly comparable to that of the protein. However, these results underscore the importance of not relying solely on an error threshold for choosing FMM parameters as universally transferable error metrics are difficult to define for MD simulations. Instead, FMM-AutoOpt provides a rapid means of characterizing the convergence behavior across the FMM parameter space.

### FMM_SPARSE Environment Variable

FMM-AutoOpt facilitates automated performance evaluations with and without the FMM_SPARSE environment variable. Employing this feature, we evaluated two different system types: a gas-phase protein in an extended conformation and water vapor (Figures 4 and S3).

**Figure 4:**
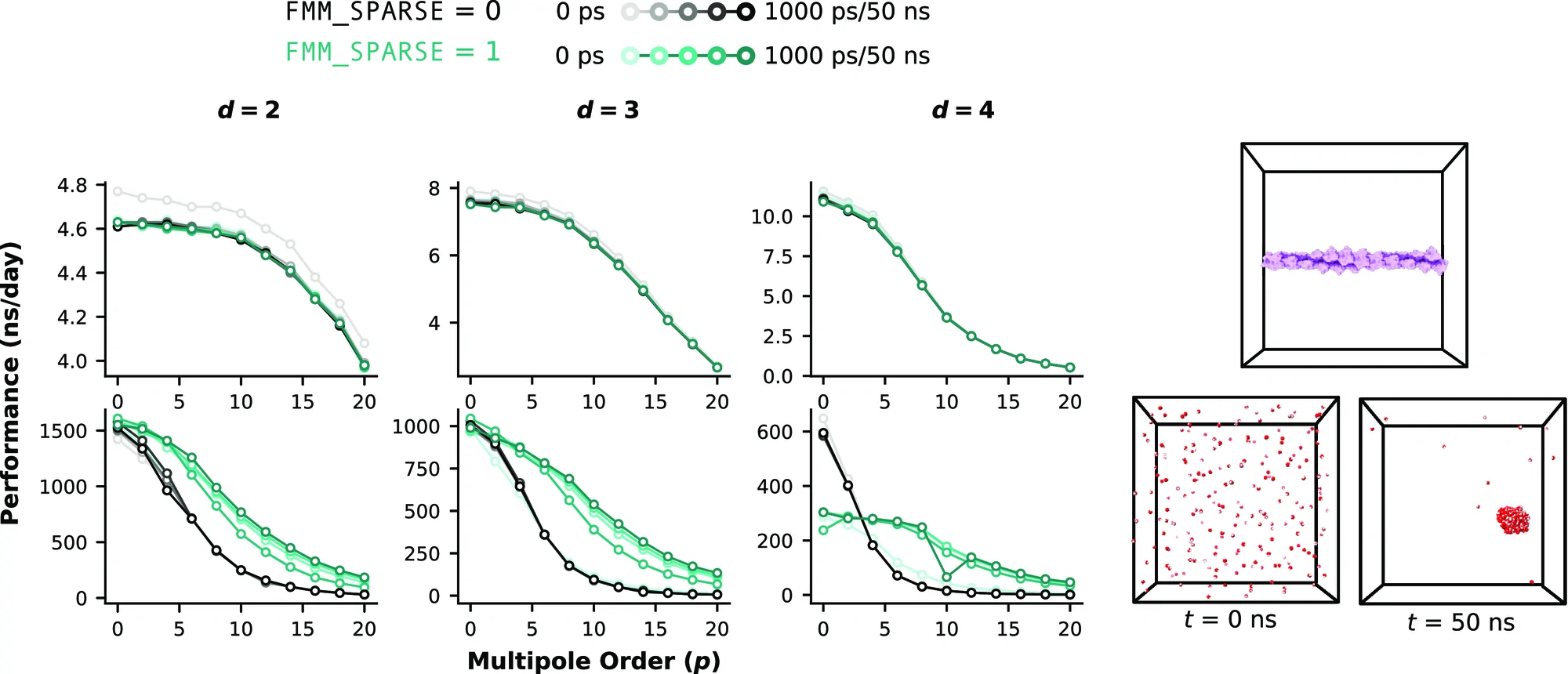
Performance benchmarks comparing FMM_SPARSE set to 0 and 1. Upper panel: protein in vacuum, with five frames sampled from a 1000 ps trajectory. Lower panel: water vapor, with five frames sampled from a 50 ns trajectory. Computational details are given in the text and corresponding accuracy data are shown in Figure S3. Snapshots of the simulated systems are shown on the right. The protein underwent minimal structural changes across the sampled frames.

The benchmarked protein was a 1 MDa muscle thin filament complex (PDB ID 5MVA^17^) simulated using default parameters and open boundary conditions. The complex has an aspect ratio of nearly 10:1, resulting in significant void space within the mandatory cubic simulation box and making it a favorable test case for the FMM_SPARSE. Interestingly, the FMM_SPARSE variable had a negligible influence on the performance. This indicates that for this system, the computational overhead associated with identifying and skipping empty octree boxes largely offsets the resulting reduction in multipole construction and FMM interactions. This also suggests that the inherent clustering of gas-phase proteins already limits the computational cost of evaluating empty regions. It should, however, be noted that the performance reported here reflects the current implementation of FMM_SPARSE on the current hardware, and future implementation optimizations may alter this trade-off.

The water vapor system comprised 216 TIP3P^15^ water molecules within a periodic cubic box with a side length of 16 nm. The configuration was generated by scaling the oxygen atom coordinates of the standard SPC216 water box supplied with GROMACS, a resource typically utilized for protein solvation via the gmx solvate utility. We performed a 50 ns simulation of the system during which a large fraction of the water molecules rapidly clustered into a droplet. Benchmarking five frames sampled evenly throughout this trajectory illustrates the utility of evaluating multiple conformations when the system undergoes substantial structural changes.

Following droplet formation, performance enhancements with FMM_SPARSE were substantial and became most pronounced at higher tree depths and at moderate multipole orders. The dependence on tree depth is expected as finer spatial partitioning increases the number of empty cells that can be skipped. Interestingly, at very high multipole orders, the relative benefit of skipping empty boxes diminishes, indicating that the computational savings become less significant, relative to the total runtime. Whether this reflects fundamental algorithmic trade-offs or characteristics of the current implementation remains an open question.

## Conclusions and Outlook

FMM-AutoOpt is a utility for automated benchmarking of GROMACS FMM parameters, specifically *d*, *p*, and FMM_SPARSE. While the default parameter set was tailored primarily toward gas-phase protein systems, we demonstrate the tool’s application across selected gas-phase and solvated test cases. We emphasize that application to distinctly different systems will require careful consideration and validation of the parameter choices.

The tool is notably efficient: a comprehensive evaluation of a 1 MDa gas-phase protein, covering *d* ∈ [2, 4] and *p* ∈ [0, 20] with a step size of 2, completes in under 2 h per input frame (single node of two Intel Xeon Gold 6130 CPUs and one NVIDIA T4 GPU). For smaller systems, such as our 216-molecule water vapor system, execution time is approximately 15 min. As our benchmark results demonstrate, computational throughput varies drastically across different (*d*, *p*) parameter combinations. This modest upfront investment in optimization is therefore rapidly recovered during production simulations requiring days or weeks of compute time, where selecting an optimal parameter set yields substantial overall performance gains.

Since error convergence is frequently achieved well below *p* = 20 for most configurations, limiting the maximum *p* value is an effective strategy for rapid screening as high-order multipoles represent the most computationally intensive portion of the parameter space. Once an appropriate error threshold has been established for a given system, the --p-stop feature can be used to terminate the *p* scan once convergence below the threshold has been reached for a given tree depth. However, the greatest strength of FMM-AutoOpt is its ability to efficiently perform complete parameter scans across multiple frames, providing users with a comprehensive view of the convergence behavior and enabling the well-informed selection of FMM parameters.

FMM offers advantages over PME for systems that are large in volume, sparse, or require open boundaries and net charges. It also has potential for efficient multinode GPU scaling, though this is not yet implemented with GROMACS FMM. Coupled with the well-established GROMACS package, we foresee an increased use of GROMACS FMM for a variety of MD simulations, where FMM-AutoOpt will be useful for ensuring accuracy while saving both researcher time and computational resources.

## Supplementary Material



## Data Availability

The FMM-AutoOpt code is freely available via GitHub: https://github.com/lpersson/FMM-AutoOpt.git.

## Notes

Benchmarking and validation of the reference calculations used for periodic boundary condition simulations; complete benchmarking data, including error and performance analyses, for all systems presented in the manuscript (PDF)
