# Supplementary material for: FMM-AutoOpt: A Python Tool for Automated GROMACS FMM Parameter Optimization
Source: J Chem Inf Model. 2026 Sep 4;66(18):11556–62. doi: 10.1021/acs.jcim.6c01211 (PMC13625960; doi:10.1021/acs.jcim.6c01211)
Supplement: Supplementary file 1 [file ci6c01211_si_001.pdf]

Supporting Information
FMM-AutoOpt: A Python Tool for Automated GROMACS FMM Parameter Optimization

Louise J. Persson*[1] and Erik G. Marklund*[1]

[1]Department of Chemistry for Life Sciences, Uppsala University, SE-75123 Uppsala, Sweden

*Email: louise.persson@kemi.uu.se, erik.marklund@kemi.uu.se

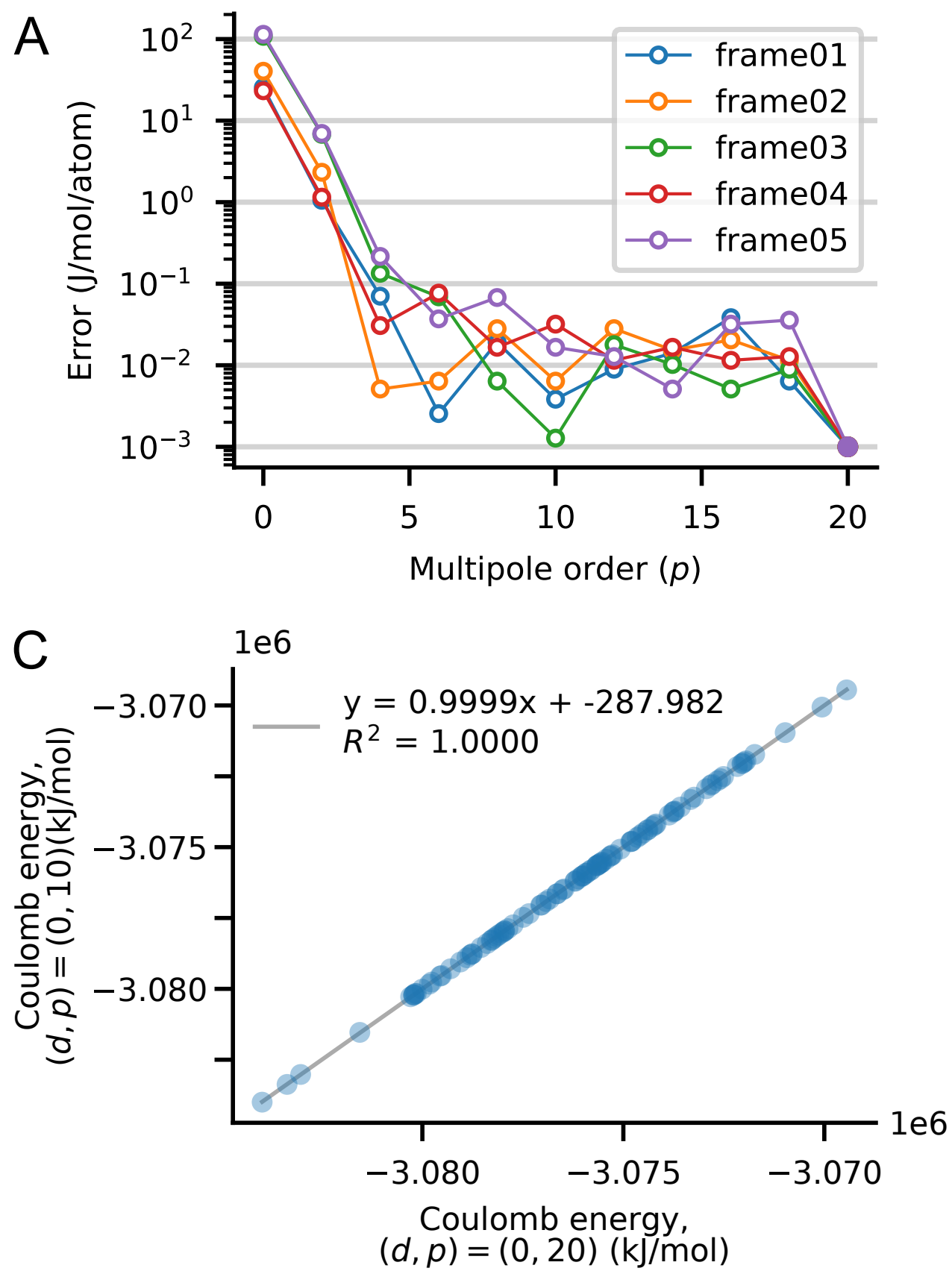

Figure S1: Validation of the reference calculations for periodic boundary condition simulations. Benchmarks were performed on the solvated protein (A, C) and water vapor (B, D) systems described in the main text. (A, B) Relative error as a function of multipole order p at a fixed tree depth $d = 0$. The error is evaluated over five frames relative to $(d, p) = (0, 20)$, representing the highest-accuracy setting computationally feasible on the current hardware. (C, D) Correlation of the total Coulomb energy evaluated at $(d, p) = (0, 10)$ versus the reference state $(d, p) = (0, 20)$ across 100 frames. The linear regression demonstrates excellent agreement between the two settings, supporting the use of $(d, p) = (0, 20)$ as the reference state.

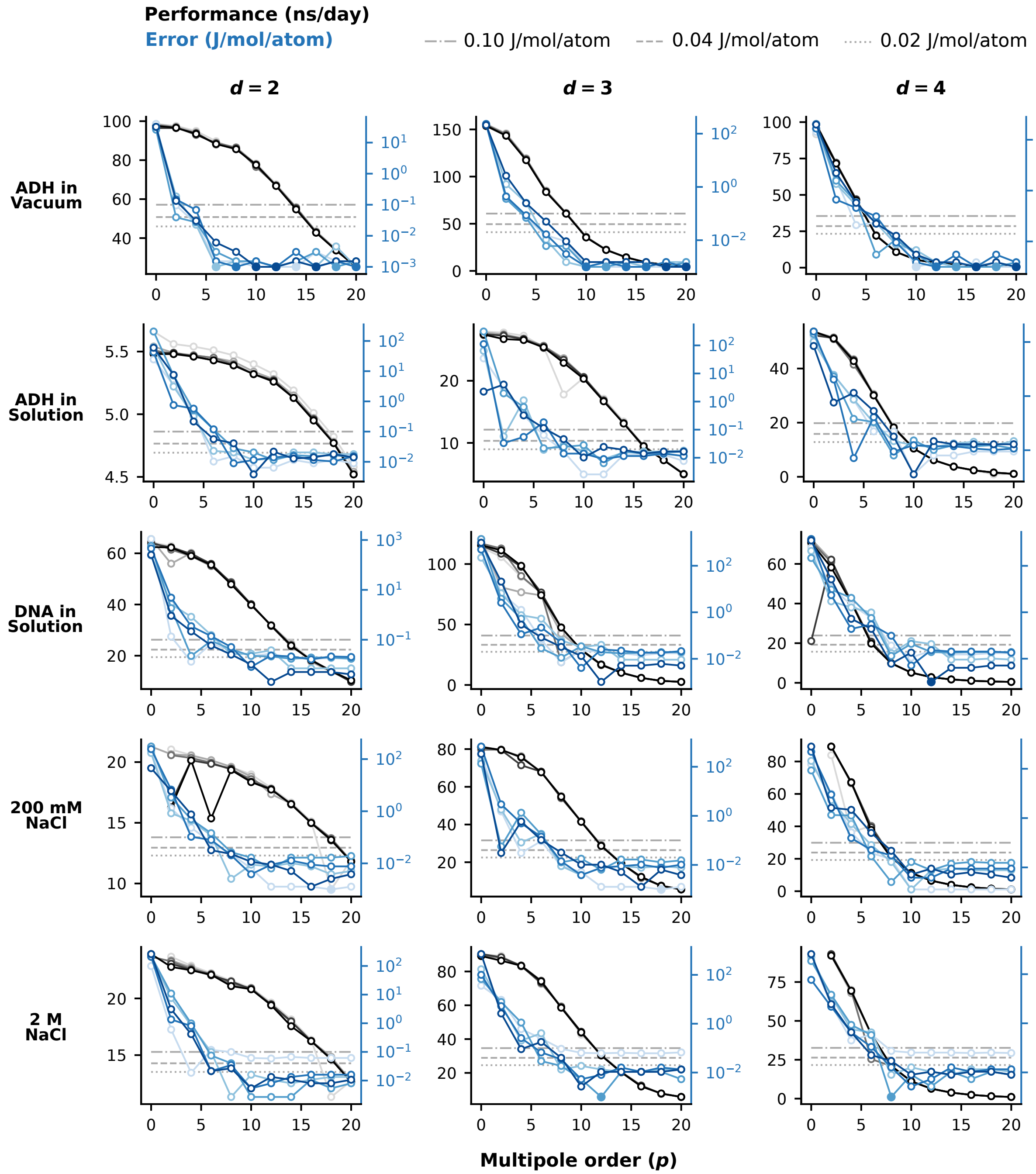

Figure S2: Performance and accuracy benchmarks of multiple solvated systems and one gas-phase protein. Each curve corresponds to a single input frame, with the lightest color representing the earliest sampled frame and the darkest the latest. Solid markers denote zero error; these are plotted at 0.001 J/mol/atom for compatibility with the logarithmic scale. Further computational details are given in the main text.

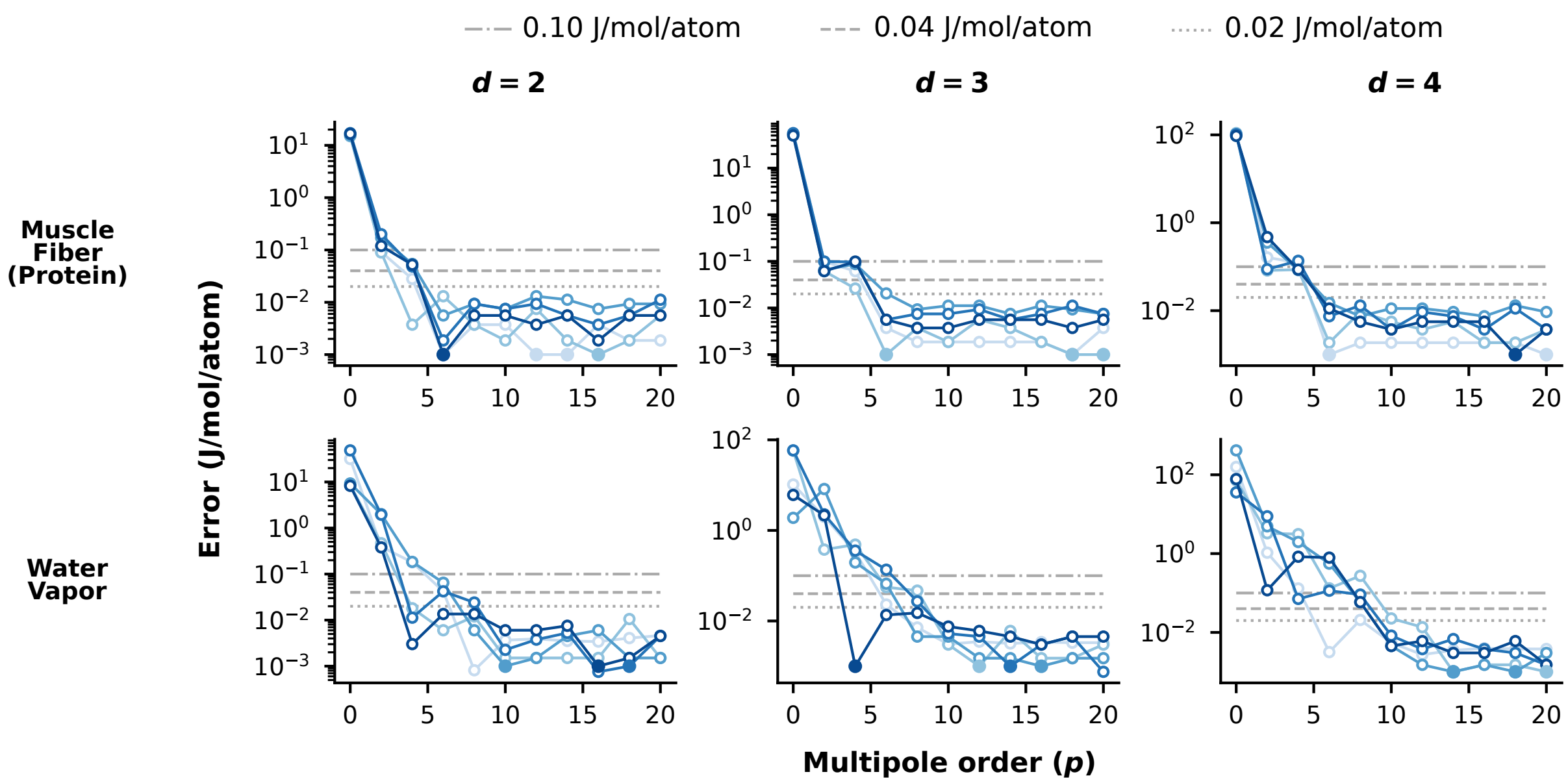

Figure S3: Accuracy benchmarks performed on two sparse systems. Because FMM_SPARSE does not affect the accuracy of the FMM calculation, FMM-AutoOpt performs the accuracy benchmark only once, with the variable set to 0. Each curve corresponds to a single input frame, with the lightest color representing the earliest sampled frame and the darkest the latest. Further computational details are given in the main text.
